# Di­aqua­{2,2′-dimeth­oxy-6,6′-[(1*E*,1′*E*)-propane-1,3-diylbis(aza­nylyl­idene)bis­(methanylyl­idene)]diphenolato}nickel(II)

**DOI:** 10.1107/S1600536813016188

**Published:** 2013-06-15

**Authors:** Amitabha Datta, Barbara Machura, Jui-Hsien Huang, Shiann-Cherng Sheu

**Affiliations:** a Department of Chemistry, National Changhua University of Education, Changhua 50058, Taiwan; b Department of Inorganic and Radiation Chemistry, Institute of Chemistry, University of Silesia, 9th Szkolna Street, 40-006 Katowice, Poland; c Department of Occupational Health and Safety, Chang Jung Christian University, Tainan City 71101, Taiwan

## Abstract

In the mol­ecule of the title compound, [Ni(C_19_H_20_N_2_O_4_)(H_2_O)_2_], the central Ni^II^ ion lies on a mirror plane and is surrounded by an N_2_O_4_ coordination set in the form of a distorted octa­hedron defined by the O atoms of two water mol­ecules and by two phenolic O and two imine N atoms of the tetra­dentate Schiff base ligand. In the crystal, O—H⋯O hydrogen bonds between the water mol­ecules and the phenolic and meth­oxy O atoms of neighbouring mol­ecules lead to the formation of rods propagating parallel to [100].

## Related literature

For related complexes with similar ligands, see: Sen *et al.* (2006 ▶); Thakurta *et al.* (2009*a* ▶,*b* ▶, 2010*a* ▶,*b* ▶).
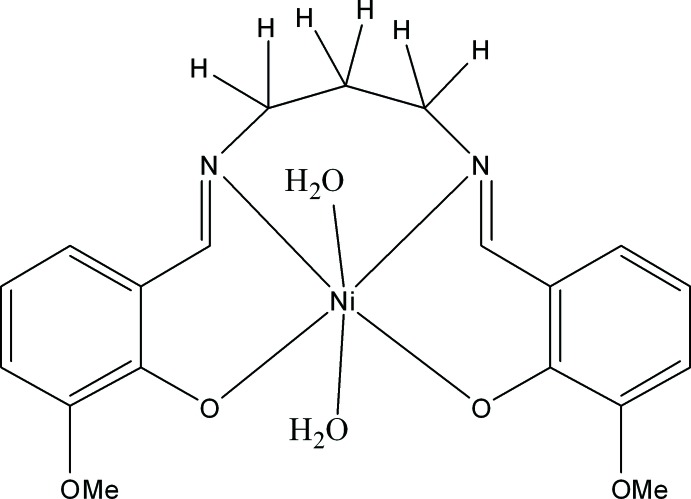


## Experimental

### 

#### Crystal data

[Ni(C_19_H_20_N_2_O_4_)(H_2_O)_2_]
*M* *_r_* = 435.11
Orthorhombic, 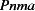

*a* = 7.4920 (2) Å
*b* = 22.1442 (6) Å
*c* = 11.6045 (3) Å
*V* = 1925.24 (9) Å^3^
*Z* = 4
Mo *K*α radiation
μ = 1.05 mm^−1^
*T* = 295 K
0.30 × 0.25 × 0.20 mm

#### Data collection

Bruker SMART CCD diffractometer
Absorption correction: multi-scan (*SADABS*; Bruker, 2007 ▶) *T*_min_ = 0.782, *T*_max_ = 1.000
20064 measured reflections
1745 independent reflections
1578 reflections with *I* > 2σ(*I*)
*R*_int_ = 0.035

#### Refinement

*R*[*F* ^2^ > 2σ(*F* ^2^)] = 0.025
*wR*(*F* ^2^) = 0.073
*S* = 1.06
1745 reflections
142 parameters
2 restraints
H atoms treated by a mixture of independent and constrained refinement
Δρ_max_ = 0.80 e Å^−3^
Δρ_min_ = −0.31 e Å^−3^

### 

Data collection: *APEX2* (Bruker, 2007 ▶); cell refinement: *SAINT* (Bruker, 2007 ▶); data reduction: *SAINT*; program(s) used to solve structure: *SHELXS97* (Sheldrick, 2008 ▶); program(s) used to refine structure: *SHELXL97* (Sheldrick, 2008 ▶); molecular graphics: *SHELXTL* (Sheldrick, 2008 ▶); software used to prepare material for publication: *publCIF* (Westrip, 2010 ▶).

## Supplementary Material

Crystal structure: contains datablock(s) I, global. DOI: 10.1107/S1600536813016188/wm2747sup1.cif

Structure factors: contains datablock(s) I. DOI: 10.1107/S1600536813016188/wm2747Isup2.hkl

Additional supplementary materials:  crystallographic information; 3D view; checkCIF report

## Figures and Tables

**Table 1:** Selected bond lengths (Å)

Ni1—O1^i^	2.0131 (13)
Ni1—O1	2.0131 (13)
Ni1—N1	2.0684 (16)
Ni1—N1^i^	2.0684 (16)
Ni1—O4	2.1048 (19)
Ni1—O3	2.113 (2)

Symmetry code: (i) 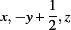.

**Table 2:** Hydrogen-bond geometry (Å, °)

*D*—H⋯*A*	*D*—H	H⋯*A*	*D*⋯*A*	*D*—H⋯*A*
O4—H4*O*⋯O2^ii^	0.83 (1)	2.49 (2)	3.1433 (19)	136 (2)
O4—H4*O*⋯O1^ii^	0.83 (1)	2.11 (2)	2.823 (2)	143 (2)
O3—H3*O*⋯O1^iii^	0.83 (1)	2.37 (2)	3.059 (2)	140 (2)
O3—H3*O*⋯O2^iii^	0.83 (1)	2.21 (2)	2.9432 (18)	147 (2)

Symmetry codes: (ii) 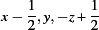; (iii) 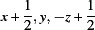.

**Fig. 1.**
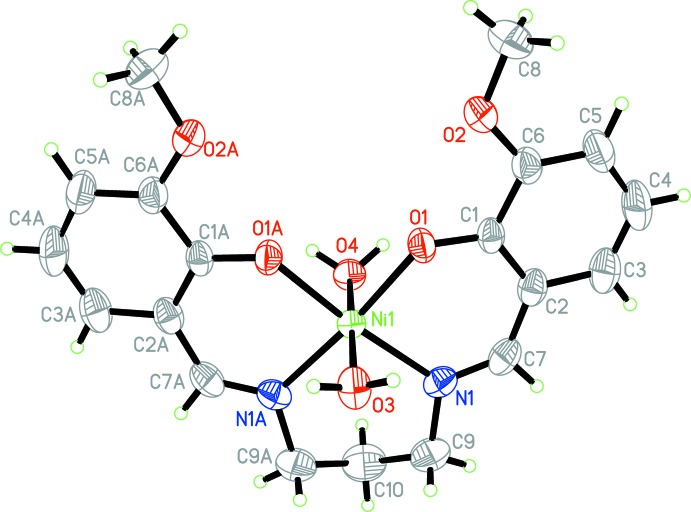
The molecular structure of the title complex, showing displacement ellipsoids at the 50% probability level. [Symmetry code: (A) x, 1/2 - y, z].

**Fig. 2.**
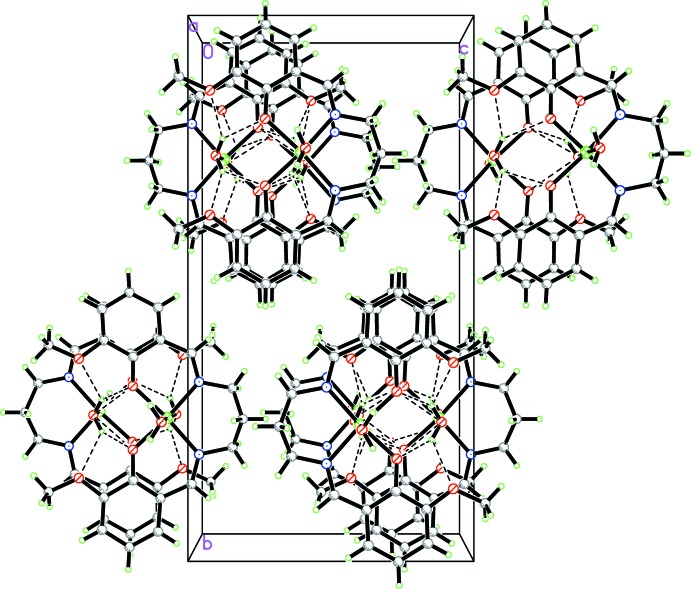
Packing diagram of the title compound as viewed down the a axis. Intermolecular O—H···O hydrogen bonds are shown as dashed lines.

## supplementary crystallographic information

### Comment

In the title compound, [Ni(C_19_H_20_N_2_O_4_)(H_2_O)_2_], the Ni(II) ion has site symmetry *m* and exhibits a distorted octahedral coordination environment defined by two water molecules and the tetradentate ligand, 6,6'-(1*E*,1'*E*)-(propane-1,3-diylbis(azan-1-yl-1-ylidene)bis(methan-1-yl-1-ylidene)bis(2-methoxyphenol) (*L*) that coordinates *via* two phenolic O and two imine N atoms (Fig. 1). The bond angles around the Ni(II) ion are slightly distorted from those of a regular octahedron and range from 85.57 (7)° to 175.62 (8)°. The two O_water_ molecules lie at the *trans* position of the octahedron. The *cis* Ni—O_phenolic_ bond lengths [2.0131 (13) Å] is considerably smaller than the *trans* Ni—O_water_ bond lengths [2.113 (2) and 2.1048 (19) Å] (Table 1). In the molecule, the dihedral angle between the (C1—C7, N1) plane and its symmetry-related counterpart (C1A—C7A, N1A) is 27.58 (7)° [A) *x*, 1/2 - *y*, *z*].

The resulting coordination geometry around the metal cation is comparable to that of complexes with similar Schiff-bases. See, for example: Thakurta *et al.* (2009**a*,b*,2010**a*,b*).

In the crystal structure of the title comound, intermolecular O—H···O hydrogen bonds between water molecules as donor groups and phenolic O and methoxy O atoms of neighbouring molecules as acceptor groups are observed (Table 2). The hydrogen bonding interactions lead to the formation of rods propagating parallel to [100] (Fig. 2).

### Experimental

The tetradentate Schiff base precursor was prepared according to the literature procedure (Sen *et al.*, 2006). To a hot methanolic solution (20 ml) of Ni(CH_3_COO)_2_^.^4H_2_O (0.248 g, 1.0 mmol), the ligand (1.0 mmol) was added, which produced immediately an intensely brown solution. The mixture was then kept at room temperature. After slow evaporation of the brown solution, dark chocolate-brown single crystals with a rectangular form were separated out in 5 days. The crystals were filtered off and washed with water and dried in air.

### Refinement

Carbon-bound H-atoms were placed in calculated positions (C—H 0.93 to 0.97 Å) and were included in the refinement in the riding model approximation. The H atoms of the water molecules were located in a difference map and were refined with an O—H distances restraint of 0.85 (1) Å.

### Crystal data

**Table:**

[Ni(C_19_H_20_N_2_O_4_)(H_2_O)_2_]	*F*(000) = 912
*M**_r_* = 435.11	*D*_x_ = 1.501 Mg m^−^^3^
Orthorhombic, *P**n**m**a*	Mo *K*α radiation, λ = 0.71073 Å
Hall symbol: -P 2ac 2n	Cell parameters from 8168 reflections
*a* = 7.4920 (2) Å	θ = 3.4–29.2°
*b* = 22.1442 (6) Å	µ = 1.05 mm^−^^1^
*c* = 11.6045 (3) Å	*T* = 295 K
*V* = 1925.24 (9) Å^3^	Rectangular, brown
*Z* = 4	0.30 × 0.25 × 0.20 mm

### Data collection

**Table:**

Bruker SMART CCD diffractometer	1745 independent reflections
Radiation source: fine-focus sealed tube	1578 reflections with *I* > 2σ(*I*)
Graphite monochromator	*R*_int_ = 0.035
phi and ω scans	θ_max_ = 25.0°, θ_min_ = 3.4°
Absorption correction: multi-scan (*SADABS*; Bruker, 2007)	*h* = −8→8
*T*_min_ = 0.782, *T*_max_ = 1.000	*k* = −26→26
20064 measured reflections	*l* = −13→13

### Refinement

**Table:**

Refinement on *F*^2^	Primary atom site location: structure-invariant direct methods
Least-squares matrix: full	Secondary atom site location: difference Fourier map
*R*[*F*^2^ > 2σ(*F*^2^)] = 0.025	Hydrogen site location: inferred from neighbouring sites
*wR*(*F*^2^) = 0.073	H atoms treated by a mixture of independent and constrained refinement
*S* = 1.06	*w* = 1/[σ^2^(*F*_o_^2^) + (0.0386*P*)^2^ + 0.966*P*] where *P* = (*F*_o_^2^ + 2*F*_c_^2^)/3
1745 reflections	(Δ/σ)_max_ < 0.001
142 parameters	Δρ_max_ = 0.80 e Å^−^^3^
2 restraints	Δρ_min_ = −0.31 e Å^−^^3^

### Special details

**Table:**

Geometry. All e.s.d.'s (except the e.s.d. in the dihedral angle between two l.s. planes) are estimated using the full covariance matrix. The cell e.s.d.'s are taken into account individually in the estimation of e.s.d.'s in distances, angles and torsion angles; correlations between e.s.d.'s in cell parameters are only used when they are defined by crystal symmetry. An approximate (isotropic) treatment of cell e.s.d.'s is used for estimating e.s.d.'s involving l.s. planes.
Refinement. Refinement of *F*^2^ against ALL reflections. The weighted *R*-factor *wR* and goodness of fit *S* are based on *F*^2^, conventional *R*-factors *R* are based on *F*, with *F* set to zero for negative *F*^2^. The threshold expression of *F*^2^ > σ(*F*^2^) is used only for calculating *R*-factors(gt) *etc*. and is not relevant to the choice of reflections for refinement. *R*-factors based on *F*^2^ are statistically about twice as large as those based on *F*, and *R*- factors based on ALL data will be even larger.

### Fractional atomic coordinates and isotropic or equivalent isotropic displacement parameters (Å^2^)

**Table:**

	*x*	*y*	*z*	*U*_iso_*/*U*_eq_	
Ni1	0.47233 (4)	0.2500	0.10284 (3)	0.02817 (13)	
N1	0.4635 (2)	0.31989 (7)	−0.01527 (14)	0.0375 (4)	
O1	0.48285 (18)	0.31175 (5)	0.22998 (11)	0.0359 (3)	
O2	0.4497 (2)	0.36489 (7)	0.42580 (13)	0.0531 (4)	
O3	0.7532 (3)	0.2500	0.08662 (18)	0.0406 (5)	
O4	0.1914 (3)	0.2500	0.10516 (17)	0.0376 (4)	
C1	0.4402 (2)	0.36858 (8)	0.22558 (17)	0.0324 (4)	
C2	0.4123 (3)	0.40152 (8)	0.12334 (18)	0.0378 (4)	
C3	0.3675 (3)	0.46364 (9)	0.1292 (2)	0.0512 (6)	
H3	0.3515	0.4853	0.0613	0.061*	
C4	0.3474 (3)	0.49203 (10)	0.2318 (2)	0.0605 (7)	
H4	0.3166	0.5327	0.2339	0.073*	
C5	0.3728 (3)	0.46043 (9)	0.3339 (2)	0.0527 (6)	
H5	0.3581	0.4799	0.4042	0.063*	
C6	0.4197 (3)	0.40052 (8)	0.33109 (18)	0.0396 (5)	
C7	0.4323 (3)	0.37529 (9)	0.01098 (18)	0.0423 (5)	
H7	0.4212	0.4018	−0.0508	0.051*	
C8	0.4391 (4)	0.39102 (12)	0.5356 (2)	0.0696 (8)	
H8A	0.5251	0.4230	0.5417	0.104*	
H8B	0.4636	0.3609	0.5930	0.104*	
H8C	0.3214	0.4070	0.5474	0.104*	
C9	0.4892 (4)	0.30703 (12)	−0.1376 (2)	0.0594 (6)	
H9A	0.6162	0.3042	−0.1531	0.071*	
H9B	0.4428	0.3406	−0.1822	0.071*	
C10	0.4007 (5)	0.2500	−0.1775 (3)	0.0624 (9)	
H10A	0.2782	0.2500	−0.1504	0.075*	
H10B	0.3976	0.2500	−0.2610	0.075*	
H4O	0.150 (3)	0.2810 (7)	0.1357 (19)	0.059 (7)*	
H3O	0.802 (3)	0.2808 (8)	0.113 (2)	0.067 (8)*	

### Atomic displacement parameters (Å^2^)

**Table:**

	*U*^11^	*U*^22^	*U*^33^	*U*^12^	*U*^13^	*U*^23^
Ni1	0.0280 (2)	0.0281 (2)	0.0284 (2)	0.000	−0.00017 (12)	0.000
N1	0.0365 (9)	0.0418 (9)	0.0342 (9)	0.0008 (7)	0.0009 (7)	0.0074 (7)
O1	0.0469 (8)	0.0247 (6)	0.0360 (7)	0.0037 (5)	−0.0038 (6)	−0.0011 (5)
O2	0.0838 (12)	0.0355 (8)	0.0401 (8)	−0.0063 (7)	0.0054 (8)	−0.0076 (6)
O3	0.0287 (10)	0.0336 (11)	0.0596 (13)	0.000	−0.0046 (9)	0.000
O4	0.0288 (10)	0.0396 (12)	0.0443 (11)	0.000	0.0044 (8)	0.000
C1	0.0260 (9)	0.0251 (8)	0.0461 (11)	−0.0024 (7)	0.0001 (8)	−0.0005 (7)
C2	0.0306 (10)	0.0315 (10)	0.0514 (12)	0.0004 (8)	−0.0014 (8)	0.0058 (8)
C3	0.0481 (13)	0.0344 (11)	0.0711 (16)	0.0043 (9)	−0.0079 (11)	0.0100 (10)
C4	0.0610 (15)	0.0280 (10)	0.093 (2)	0.0089 (10)	−0.0026 (13)	−0.0029 (12)
C5	0.0551 (13)	0.0333 (11)	0.0698 (16)	−0.0011 (9)	0.0075 (11)	−0.0142 (10)
C6	0.0383 (10)	0.0310 (10)	0.0494 (12)	−0.0057 (8)	0.0040 (9)	−0.0036 (8)
C7	0.0406 (11)	0.0406 (11)	0.0457 (12)	0.0008 (9)	−0.0037 (9)	0.0154 (9)
C8	0.099 (2)	0.0630 (16)	0.0469 (14)	−0.0099 (15)	0.0072 (13)	−0.0182 (12)
C9	0.0818 (18)	0.0607 (16)	0.0357 (12)	0.0025 (13)	0.0073 (11)	0.0096 (11)
C10	0.076 (2)	0.077 (2)	0.0342 (17)	0.000	−0.0113 (16)	0.000

### Geometric parameters (Å, º)

**Table:**

Ni1—O1^i^	2.0131 (13)	C3—C4	1.355 (3)
Ni1—O1	2.0131 (13)	C3—H3	0.9300
Ni1—N1	2.0684 (16)	C4—C5	1.389 (3)
Ni1—N1^i^	2.0684 (16)	C4—H4	0.9300
Ni1—O4	2.1048 (19)	C5—C6	1.373 (3)
Ni1—O3	2.113 (2)	C5—H5	0.9300
N1—C7	1.286 (3)	C7—H7	0.9300
N1—C9	1.461 (3)	C8—H8A	0.9600
O1—C1	1.299 (2)	C8—H8B	0.9600
O2—C6	1.372 (3)	C8—H8C	0.9600
O2—C8	1.402 (3)	C9—C10	1.500 (3)
O3—H3O	0.830 (10)	C9—H9A	0.9700
O4—H4O	0.833 (9)	C9—H9B	0.9700
C1—C2	1.408 (3)	C10—C9^i^	1.500 (3)
C1—C6	1.422 (3)	C10—H10A	0.9700
C2—C3	1.418 (3)	C10—H10B	0.9700
C2—C7	1.435 (3)		
			
O1^i^—Ni1—O1	85.57 (7)	C3—C4—C5	120.0 (2)
O1^i^—Ni1—N1	174.34 (6)	C3—C4—H4	120.0
O1—Ni1—N1	88.78 (6)	C5—C4—H4	120.0
O1^i^—Ni1—N1^i^	88.78 (6)	C6—C5—C4	120.1 (2)
O1—Ni1—N1^i^	174.34 (6)	C6—C5—H5	119.9
N1—Ni1—N1^i^	96.88 (9)	C4—C5—H5	119.9
O1^i^—Ni1—O4	91.71 (6)	O2—C6—C5	125.4 (2)
O1—Ni1—O4	91.71 (6)	O2—C6—C1	112.71 (16)
N1—Ni1—O4	88.65 (6)	C5—C6—C1	121.9 (2)
N1^i^—Ni1—O4	88.65 (6)	N1—C7—C2	128.35 (18)
O1^i^—Ni1—O3	91.51 (6)	N1—C7—H7	115.8
O1—Ni1—O3	91.51 (6)	C2—C7—H7	115.8
N1—Ni1—O3	88.44 (6)	O2—C8—H8A	109.5
N1^i^—Ni1—O3	88.44 (6)	O2—C8—H8B	109.5
O4—Ni1—O3	175.62 (8)	H8A—C8—H8B	109.5
C7—N1—C9	116.12 (18)	O2—C8—H8C	109.5
C7—N1—Ni1	124.25 (14)	H8A—C8—H8C	109.5
C9—N1—Ni1	119.60 (15)	H8B—C8—H8C	109.5
C1—O1—Ni1	128.27 (12)	N1—C9—C10	113.9 (2)
C6—O2—C8	118.81 (18)	N1—C9—H9A	108.8
Ni1—O3—H3O	113.9 (19)	C10—C9—H9A	108.8
Ni1—O4—H4O	112.4 (17)	N1—C9—H9B	108.8
O1—C1—C2	124.84 (17)	C10—C9—H9B	108.8
O1—C1—C6	118.32 (17)	H9A—C9—H9B	107.7
C2—C1—C6	116.85 (16)	C9^i^—C10—C9	114.7 (3)
C1—C2—C3	119.82 (19)	C9^i^—C10—H10A	108.6
C1—C2—C7	122.70 (17)	C9—C10—H10A	108.6
C3—C2—C7	117.45 (19)	C9^i^—C10—H10B	108.6
C4—C3—C2	121.2 (2)	C9—C10—H10B	108.6
C4—C3—H3	119.4	H10A—C10—H10B	107.6
C2—C3—H3	119.4		

Symmetry code: (i) *x*, −*y*+1/2, *z*.

### Hydrogen-bond geometry (Å, º)

**Table:**

*D*—H···*A*	*D*—H	H···*A*	*D*···*A*	*D*—H···*A*
O4—H4*O*···O2^ii^	0.83 (1)	2.49 (2)	3.1433 (19)	136 (2)
O4—H4*O*···O1^ii^	0.83 (1)	2.11 (2)	2.823 (2)	143 (2)
O3—H3*O*···O1^iii^	0.83 (1)	2.37 (2)	3.059 (2)	140 (2)
O3—H3*O*···O2^iii^	0.83 (1)	2.21 (2)	2.9432 (18)	147 (2)

Symmetry codes: (ii) *x*−1/2, *y*, −*z*+1/2; (iii) *x*+1/2, *y*, −*z*+1/2.
